# Editorial: Citizenship and democratization: perspectives from different gender-theoretical approaches

**DOI:** 10.3389/fsoc.2023.1343538

**Published:** 2024-01-05

**Authors:** Jana Günther, Eva Maria Hinterhuber

**Affiliations:** ^1^Evangelische Hochschule Darmstadt, Darmstadt, Germany; ^2^Faculty Society and Economics, Rhine-Waal University for Applied Sciences, Kleve, Germany

**Keywords:** citizenship, gender relations, democratization, women's movement, crisis

The year 1918 was significant in many ways, as it marked the end of the First World War. At the same time, the impact, and upheaval of this event enabled civil society activists and politically institutionalized actors in European countries to pick up the threads of democratic social movements and parliamentary aspirations and use “political opportunity structures” (Marx-Ferree and McClurg Mueller, 2006, 39–45) to achieve civil rights for larger sections of the population. In addition to entirely new forms of political participation, new state social laws and the improvement of co-determination rights for larger population groups, peace and more freedom led to (new) social movements and forms of protest. National women's movements in particular were successful before the First World War, were limited in their possibilities during the war and reorganized after the First World War (Offen, 2000, 272–356).

One result of this process—albeit with differences between European states—was that more social groups gained the right to vote, and new social rights and expanded access to education and gainful employment for the working class and women of all classes were achieved; in that regard, the cloud indeed had a silver lining. But still: After the First World War, European countries were plagued by social and political problems. The interwar period was therefore not only a time of opportunity, but also of crisis. The Weimar Republic is a good example of how an open, inclusive, and diverse culture like the “Roaring Twenties” can go hand in hand with increasing political destabilization and social resignation. The year 1923^1^ shows how thin the line can be between political and social disruption and actual change. Attacks on gender rights as an sign of violent social conditions (Roth et al., 2022, p. 9) shape state policy and law and thus have a direct impact on gender relations. The ups and downs of the decade “between the wars” are striking: more democratization on the one hand and economic collapse and inflation, growing fascism, and annexation in various parts of Europe on the other. All these developments have had an impact on women's and family policies, sometimes in the mode of two steps forward and three steps back. Political and social change affects gender relations, but both are a product of social movements, as other examples around the world show.

In 100 years later, we are facing similar difficulties such as the global financial crisis, the global care crisis, pandemics, new fascist movements and state policies, terrorist threats and ongoing wars, to name but a few of the challenges. Even today, the success of social movements goes hand in hand with counter-movements: Their scope for action is shaped by a re-dichotomization of world relations (Ruppert and Scheiterbauer, 2022) and by powerful discourses—discourses that make authoritarianism acceptable through new far-right parties in European countries, who contribute to the deterioration of democratic gender relations by linking narratives on people, gender, and migration (Wilde, 2022).

The issue “*Citizenship and democratization: perspectives from different gender-theoretical approaches*” focuses on various issues of gender, women's movements, democratization, power relations in times of civic crisis and the potential for change in different areas. In the Research Topic, analyses of historical women's movements at the beginning of the 20^th^ century illustrate the struggle for full citizenship for women. They include the study of outstanding personalities like Louise Otto-Peters in Germany's women's suffrage movement (Schötz) as well as the analysis of how women's suffrage was specifically addressed in an Austrian Social Democratic journal (Bargetz). Others discuss the significance of certain groups as the French Red Cross Ladies in the international exhibitions from 1867 to 1937 (Belliard) or provide international comparisons, here between the historical British and Russian women's movements and their stances to various forms of power (Hinterhuber and Günther). All of the contributions demonstrate the strength of the actors, individually and collectively, in oppressive power relations and in the face of constant devaluation of themselves and their concerns. At the same time, they also draw attention to hierarchies and tensions within women's movements and between movements of that time, thus underlining the relevance of intersectional approaches.

Other contributions deal with the ongoing struggle for gender equality in the context of the “third wave of democratization” (Huntington, 1993). In Poland, for example, in the mid-90s the struggle over “Polish” gender regimes gained an international dimension at the 4^th^ UN World Conference on Women in Bejing (Ramme). In Chile, the analysis reveals that, with regard to gender relations, in transition to democracy authoritarian continuities persisted (Graf). And, for post-conflict Kosovo, the strategies of the women's movement in reaching gender-responsive governance are analyzed (Holzner). Altogether, the contributions show in all clarity that democratization does not automatically go hand in hand with the democratization of gender relations.

In addition, what has been achieved once, does not remain achieved forever. Counter-movements have been gaining momentum since 2010 at the latest. For the present, the articles shed light on threats to democracy and the egalitarian welfare state, to anti-democratic claims to citizenship and gender relations—last but not least with the rise of the far-right in the Scandinavian context (Finnsdottir and Hallgrimsdottir). In other cases, such as in Russia, democratization has meanwhile given way to an authoritarian system; the article focuses on motherhood penalty, identifying the connection between a gender-specific division of labor and its impact on mothers' pensions and poverty in old age (Kingsbury).

The necessity of an intersectional approach already visible in the historical examples is emphasized again for the present, in the light of an evaluation of representation and responsiveness of the United Nations Commission on the Status of Women, revealing that it does not sufficiently include the diverse women populations worldwide (Rincker et al.).

In times of polycrisis, the rise of authoritarianism, economic upheavals, wars and the increasingly evident climate crisis cast long shadows. Democratization processes are not irreversible, they prove to be never-ending processes. Gender policy achievements and ideals in particular are becoming the focus of conflict, and their rejection is seen as the “symbolic glue” (Petö, 2015, p. 126) that holds opposing anti-democratic forces together. This poses major challenges for women's and gender policy movements. At the same time, they are acknowledged as crucial actors and potentially countervailing powers to anti-democratic and anti-gender backlashes, holding the chance that every cloud does indeed have a silver lining.

## Author contributions

JG: Writing—original draft. EH: Writing—original draft.

## Footnotes

^1^In German history, the year 1923 is often referred to as the “year of the Crisis” (Krisenjahr) (Jones, 2022) due to the immense hyperinflation and the Ruhr invasion.
